# The benefits of learning movement sequences in social interactions

**DOI:** 10.3389/fpsyg.2022.901900

**Published:** 2022-08-09

**Authors:** Guy Nahardiya, Andrey Markus, Rotem Bennet, Simone G. Shamay-Tsoory

**Affiliations:** ^1^Department of Psychology, University of Haifa, Haifa, Israel; ^2^The Integrated Brain and Behavior Research Center (IBBRC), Haifa, Israel

**Keywords:** synchrony, movement, learning, consolidation, dyadic interaction

## Abstract

Although we frequently acquire knowledge and skills through social interactions, the focus of most research on learning is on individual learning. Here we characterize Interaction Based Learning (IBL), which represents the acquisition of knowledge or skill through social interactions, and compare it to Observational Learning (OL)—learning by observation. To that end, we designed a movement synchronization paradigm whereby participants learned Tai-Chi inspired movement sequences from trained teachers in two separated sessions. We used a motion capture system to track the movement of 40 dyads comprised of a teacher and learner, who were randomly divided into OL or IBL groups, and calculated time-varying synchrony of three-dimensional movement velocity. While in the IBL group both the learner and the teacher could see each other through a transparent glass, in the OL group dyads interacted through a one-way mirror, such that the learners observed the teacher, but the teacher could not see the learners. Results show that although the number of movements recalled was not different between groups, we found improved movement smoothness in the IBL compared to the OL group, indicating movement acquisition was better in the IBL group. In addition, we found that motor synchronization levels in dyads improved over time, indicating that movement synchronization can be learned and retained. In the first session, the IBL group, but not the OL group, showed a significant improvement in synchronization. This suggests that dyadic interaction is important for learning movement sequences, and that bidirectional communication of signals and mutual feedback are essential for the consolidation of motor learning.

## Introduction

Although we acquire languages and motor skills and learn about the social world by interacting with other individuals, most models of learning are limited to understanding the acquisition of skills and knowledge of participants in socially decontextualized setting (Schilbach et al., 2013; Shamay-Tsoory and Mendelsohn, 2019). Studies that did examine learning in social context had almost exclusively focused on observational learning (OL), or vicarious learning, the learning that occurs through observing the behavior of others (Bandura and Walters, 1977; Olsson et al., 2018). In OL the learner is acquiring a skill by observation with no social interaction between learner and teacher (Bandura and Walters, 1977). This type of learning can be found in observational fear learning (Olsson and Phelps, 2007), in online courses (Joksimović et al., 2015) or when acquiring knowledge from video recordings, such as YouTube and television. Although many studies examined OL, only few studies focused on the learning that emerges from engaging in social interactions. The term Interaction-based learning (IBL) was recently coined to represent the acquisition of knowledge or skill through social interactions (Shamay-Tsoory, 2021). IBL occurs in all types of social interactions involving bidirectional exchange of information, including teacher-learner interaction, whereby one is the informed partner who transfers the information to an uninformed individual (teaching math in class), and learner-learner interactions where both partners exchange information on an equal basis (such as a dialog between friends).

The main difference between IBL and other types of learning, such as OL, or learning with partial feedback (e.g., Wu et al., 2011), is that immediate direct feedback from the teacher is available to the learner and vice versa. It is well-established that feedback between the teacher and the learner significantly increases the capacity for correcting mistakes as well as monitoring and improving the performance of a learned skill (Wu et al., 2011). Although previous research supports the notion that the interaction between learner and teacher facilitates various types of learning including language (Kuhl et al., 2003) and skill acquisition (Tauber, 1997; Bjorklund et al., 2004), there is no evidence regarding the advantage of IBL in motor learning. While it is possible that the teacher’s role is merely to demonstrate a perfect movement, as is the case in OL, it is highly likely that a bidirectional interaction between the teacher and learner, such as occurs in IBL, may contribute to better learning outcomes by permitting bidirectional synchronization to occur within the interaction. In IBL, the feedback is bidirectional. The teacher receives feedback from the learner on how the information was communicated and the learner receives feedback from the teacher on their performance. Critically, bidirectional feedback may allow the development of alignment between partners. Social alignment is the coordination of behavior over time that occurs on multiple levels, including movement synchrony, emotional and cognitive alignment (Shamay-Tsoory et al., 2019). The tendency toward alignment during social interactions may be rooted in the reward associated with bonding (Feldman, 2007; Cacioppo and Cacioppo, 2012; Atzil et al., 2018) and is evident in many types of behaviors found in the animal kingdom, involving coordinated actions, emotions and cognitions. Movement synchronization is a core component of social alignment. It involves moment-to-moment mutual adjustment of body motion (Boker and Laurenceau, 2007; Pikovsky and Rosenblum, 2007; Helm et al., 2012). Synchronization can be unconscious and spontaneous, such as clapping hands or tapping one’s feet (Kawasaki et al., 2013), or intentional, such as marching or dancing (Richardson et al., 2007). Synchronization is also assumed to be crucial for learning, especially for the learning of motor skills by imitation. While learning a motor skill by imitation can significantly contribute to the learning process (Garcia-Retamero et al., 2009), synchronization between the teacher and learner can allow moment to moment feedback and attunement of learning. The emergence of synchronization during skill learning may facilitate information exchange between individuals involved in the learning. This suggests that seeking to achieve behavioral synchronization between teacher and learner may optimize motor learning and therefore learning may be better in IBL compared to. OL.

**Figure 1:**
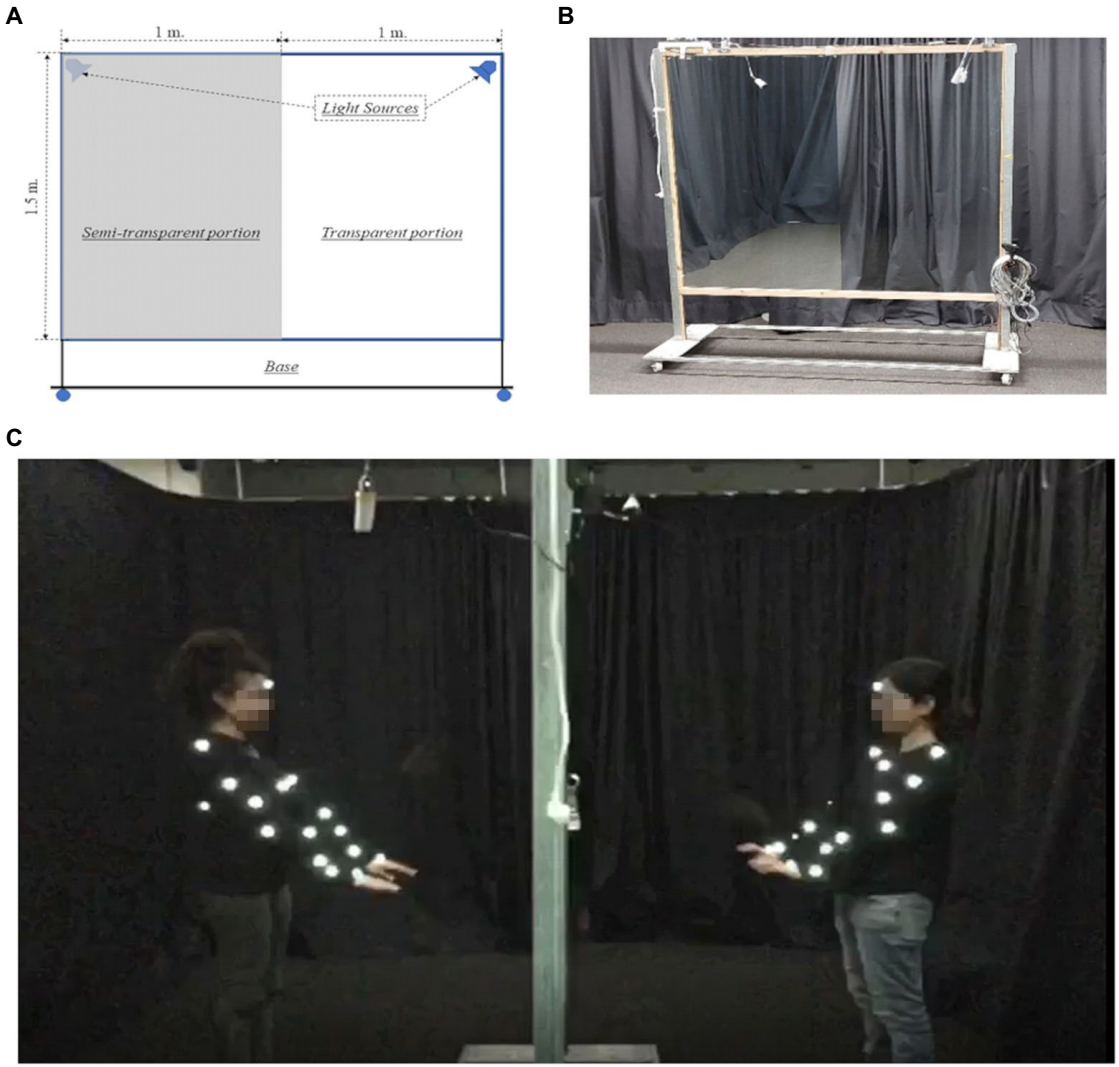
One-way mirror setup, using a semi-reflective 1 × 1.5-m mirror or a transparent 1 × 1.5-meter glass and dynamic lighting, which allowed both the Observational Learning (OL) and the Interaction Based Learning (IBL) groups to undergo training under near-identical conditions. **(A)** Illustration of the setup, showing one side of the setup (1 × 1.5 m.) as semi-transparent, and the other side (similar in size) as ordinary transparent glass, as well as the placement of the lighting. **(B)** The construction of the setup, clearly showing the difference between the sides, as previously described. **(C)** The setup used in the experiment, showing the locations of the markers.

To examine whether IBL is beneficial for motor learning, we propose an interpersonal approach that allows comparison of movement synchronization in IBL vs. OL. To that end, we designed a novel paradigm that involves the learning of movement sequences in dyads. We focused on motor learning, as it is the most basic way that individuals interact with the world (Wolpert et al., 2001). It was shown that movement synchronization is a good measure of social relationships (Yun et al., 2012), indicating that it is a core feature of interpersonal connection. Interpersonal movement synchronization is typically used to teach sports, dance, and martial arts. In our study, we probed the acquisition of Tai-Chi inspired movement sequences. Tai-Chi is a Chinese martial art which combines sequences of slow movements, requiring extensive practice with a trained teacher (Yan, 1998). Contrary to studies which used kinematically simple movement sequence paradigms, such as simple finger-tapping tasks or finger opposition sequence (FOS; e.g. Kami et al., 1995; Gabitov et al., 2015), here we used complex whole-body three-dimensional (3D) movements, which are closer to real-life, everyday behavior. In addition, measuring 3D movement allows measuring movement smoothness, which is an accepted measure of learning outcome (Sosnik et al., 2004). Nonetheless, these motions are still sufficiently constrained as to allow for accurate recording under laboratory conditions. The movements were captured and recorded using motion tracking system. To compare the OL and IBL groups we created a setup which consisted of a transparent glass and a one-way mirror. While in the one-way mirror condition, the learner could see the teacher, but the teacher could not see the learner (OL), in the transparent glass condition, both the teacher and the learner could see each other (IBL). We were thus able to create a set-up which allowed recording motion in OL and IBL conditions, under otherwise nearly identical circumstances (see Figure 1).

The first aim of the study was to examine whether learning outcomes are better in IBL vs. OL condition. In addition, we sought to examine if interpersonal movement synchronization is a skill that can be learned and retained over time. To that end, participants were trained in Tai-Chi inspired movement sequences in two separate sessions. We predicted that, overall, participants will improve their ability to mutually adapt their movements and that the IBL group will show greater incremental mutual adaption of their movements to one another and better retention of the movement synchronization, as compared to the OL group. Critically, since we hypothesized that bidirectional signals are essential for learning, we hypothesized that the IBL group will show better learning outcome in terms of movement smoothness and number of movements recollected. This was examined in a movement recollection test performed during a third session and by testing changes in movement smoothness.

## Materials and methods

### Participants

#### Exclusion criteria

Neither the designated teachers, nor the learners, had any medical conditions that might impair fine motor performance, such as learning disabilities or ADHD, neurological, psychiatric, or medical disorders. None reported any chronic medication use that could impair fine motor performance, any skeletal or muscle disease, or serious sensory or motor impairments.

#### Teachers

Two healthy women (age: 25 and 31 years), blind to the research hypothesis, were recruited and assigned as teachers. Both teachers underwent training on the Tai-Chi-inspired movement sequences that were chosen for the study prior to the beginning of the study proper. The training consisted of 5 sessions total: 3 sessions were dedicated to learning the movements and training, and 2 more for training only. Each session’s duration was about 60 min. The teachers were instructed to practice the movements on their own as well between sessions. Both teachers were right-handed.

#### Learners

Forty healthy women, aged 18 to 37, were randomly divided into one of the two learning condition groups (see below) and trained in basic Tai-Chi inspired movement sequences. Potential participants were recruited through advertisements at the University of Haifa campus and in social media. The study was approved by the Ethics Committee of University of Haifa.^1^ Informed consent was obtained prior to the experiment. Participants were right-handed. Participants were excluded if they reported having prior knowledge of Tai-Chi or martial arts which were Tai-Chi based or related.

### The transparent window (IBL) vs. one-sided mirror (OL) paradigm

Both groups performed the task in the same room. Each participant was taught by the same teacher throughout both of their training sessions. Both the OL and the IBL groups performed the learning task in the same room using the same apparatus. In both cases, the teacher and the learner were separated by a configurable one-way mirror, approx. 2 × 1.5-meter in size (Figure 1). This mirror was made such that one side of it (1 × 1.5 m.) was semi-transparent under the right lighting conditions, and served as a unidirectional mirror in the OL condition. The other side, similar in size, consisted of ordinary transparent glass, and was used in the IBL condition. Thus, both the OL and the IBL groups were able to undergo training under near-identical conditions, the main difference being that for the OL group, the lighting and mirror were configured so that the teachers could not see the learners, whereas in the IBL condition, each member of the dyad could see the other. The learners in the OL group were not made aware of the unidirectionality of the mirror in order to ensure the similarity between the conditions for both groups.

The experiment consisted of two training sessions and one testing session. In the first training session, the participants were randomly assigned into either the OL or IBL groups. They were then trained in a set of four blocks of four movements each, had a short break, and then were trained in a second set. The second training session took place 24–48 h after the first, and consisted of a similar learning procedure. The movements constituting each of the blocks within each set throughout the first and second sessions were counterbalanced as described in the next section. In the third session (testing) session, which took place 24–48 h after the second session, the participants were asked to freely recall as many movements as they could, and to re-create them as accurately as they could. The teacher was not present in this session, and there was no mirror/window. Participants were instructed not to practice the task between the meetings.

### Motor-skill learning: Tai-Chi

**Figure 2:**
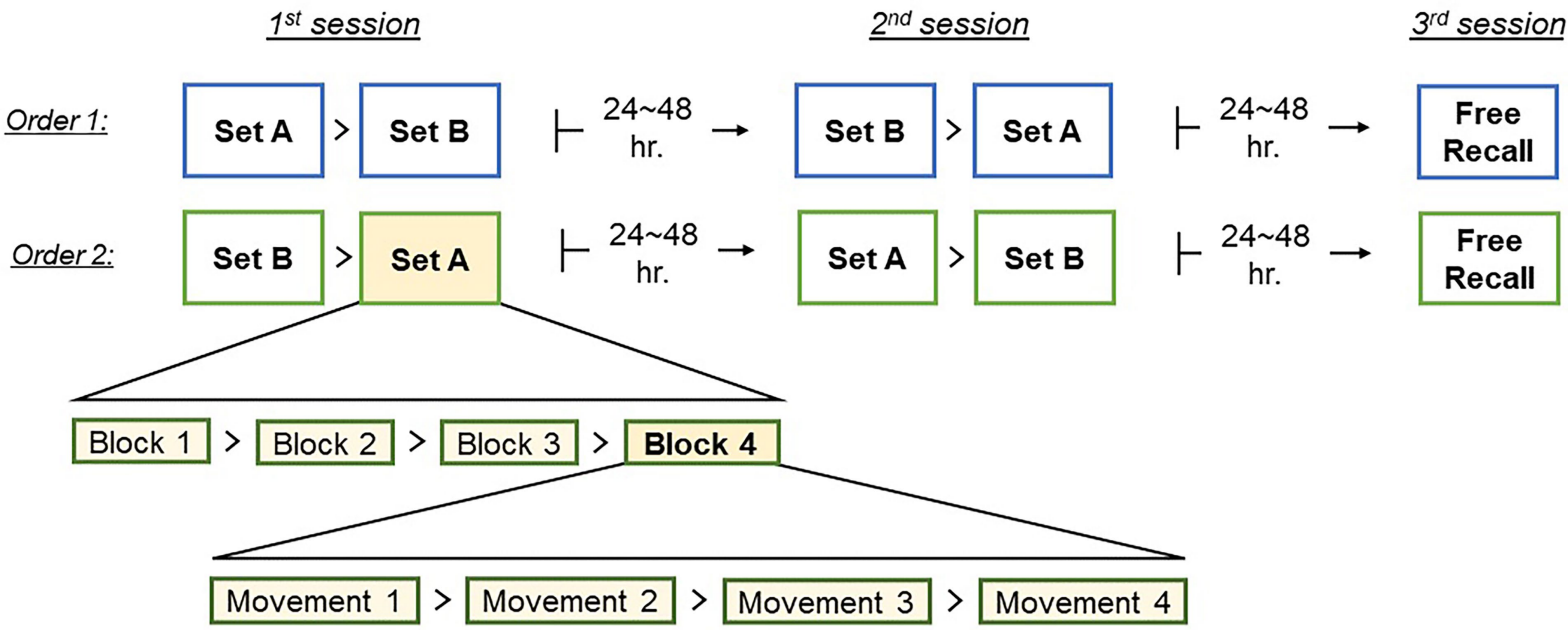
Experiment timeline: The two possible orders of learning the two sets, with examples of block order within each set, and movements order within each block. The order of movements within each block was varied within each set.

A total of eight basic, short Tai-Chi movements were used in this study. The movements were divided into two motion sets, designated as A and B. The sets were counterbalanced with respect to the perceived difficulty of the constituent motions. Each participant was exposed to both sets, in counterbalanced order: half of the participants began with set A in the first session and started with set B in the second session (A-B → B–A) and the other participants trained in the reversed order (B–A → A–B; Figure 2). The assignment of set orders to participants was counterbalanced orthogonally to the assignment to learning condition, so that each learning group (OL/IBL) had an equal number of participants with each presentation order. Furthermore, to ensure the learners’ engagement with the task, and in order to overcome any serial effects in the recall session, such as recency and/or primacy effect (Deese and Kaufman, 1957), the order of movements within each block was varied, such that no participant was exposed to any unique movement order (block) more than once.

### Motion recording

The participants’ movements were captured using the Qualisys motion-capture system. This system uses a series of synchronized high-speed infra-red cameras and optical sources to capture the positions of multiple reflective markers, which are placed on the participants’ bodies at pre-determined locations (Figure 1C). The locations of these markers were selected in accordance with Qualisys’ recommendations, so as to allow for a partial body model reconstruction from the recorded data. The participants, and their respective markers, were thus digitally captured by cameras from different angles at a rate of 100 fps. These raw recordings were then analyzed using the Qualisys motion analysis software (version 2019.3.4780). This software uses the raw recordings of the marker positions to produce the spatial location (*x*, *y*, *z* coordinates) of each marker at the time each frame was taken, with a temporal error of less than a msec, and spatial error of less than 1 mm on average (Carter et al., 2015). The software is also capable of auto-completing missing data points using mathematical interpolation. Finally, each captured marker is identified as belonging to a given body part of a given participant. Overall, the Qualisys system provided us with the spatial locations of 19 markers for each member of a teacher-learner dyad throughout each of the two training sessions, at 10 ms intervals. These data series were used in motion synchrony analyses, as described below.

### Motor-skill learning: Duration and instructions

During instruction of each movement block within a set, the teachers preform each single movement for a duration of about 10 s with about 3 s of break between movements. Breaks of about 1–2 min were given between blocks, and a break of approximately 5 min between sets. The learners were instructed to learn the movement sequences by copying the teacher’s movement as accurately as possible in a mirrored stance—i.e., to follow the motions of the teacher’s right hand with their left hand and vice versa. No verbal communication between the teachers and the learners was permitted during the learning blocks. The teachers had no prior acquaintance with the learners, and they were initially introduced to each other by the experimenter, at the onset of the first session. Some verbal communication between the teachers and the learners was reported during the breaks between the blocks, but the teachers were under strict instructions not to divulge any information related to the experiment to the learners.

### Behavioral synchrony analysis

Two pairs of markers were selected for analysis, as being the most representative of the movements used in the current study: the marker placed on the teacher’s right palm was paired with the one placed on the learner’s left palm, and vice versa. These specific markers were chosen because the palms had the highest degree of motion throughout the experiment. Both the teachers and the learners were required to remain relatively stationary at their positions adjacent to the mirror, but there were no restrictions of hand motions.

A dyadic 3D motion-sync level between the participants’ opposite palms was measured using a sliding-window Cosine of Velocity Vectors (CVV) calculation (Reiss et al., 2019). Synchrony between participants’ motions was measured by calculating the cosine of the angle between 3D velocity vectors (CVV) across different time lags (Reiss et al., 2019). This cosine is given by $C_{i,j}\left(t\right)=\frac{<v_{i}\left(t\right),v_{j}\left(t\right)>}{\left|v_{i}\left(t\right)\right|\left|v_{j}\left(t\right)\right|}$, where *t* is the time point, <*v_i_v_j_* > is the inner product of the velocities of the two participants, and |*v_i_*|and|*v_j_*| are the magnitudes of the velocities of the *i* participant and the *j* participant, respectively. CVV values ranged between 1 and –1, where CVV = 0 suggests there is no alignment in the vector directions, whereas CVV = 1 indicates that the two vectors point in the exact same direction reflecting identical motion, and CVV = −1 indicates that the two vectors point in opposite directions, reflecting mirror-like motion (e.g., when the participants are either moving toward each other or away from each other).

In the current experiment, with the learner and the teacher facing each other through the mirror, the relative directions of synchronized motion were dependent on the axis of the motion. Considering a hypothetical straight line between the two participants, co-directional motion along the two axes orthogonal to that line (i.e., left–right and up-down from each participant’s perspective) would represent best synchrony. Conversely, on the axis parallel to that imaginary line (i.e., toward or away from the other participant), motion in opposite directions would represent best synchrony. This division to axes relative to the mirror was difficult to align with the overall coordinate system given by Qualisys. Therefore, we considered both movement in the same direction and movement in opposite directions to be indicative of synchronization, and used the absolute value |CVV| as our measure of interpersonal synchrony at varying time windows. The absolute CVV of each dyad was calculated at each time point. Windowed cross-correlation between the two CVV time-series was computed for each condition, smoothed over a sliding window size of 100 samples (2 s), and computed for all the lags within a range of +/− 750 ms (in steps of 10 ms). A threshold of |CVV| > 0.35 was used to identify moments of high motion correlation, i.e., highly similar motion, either synchronized or with some lag between the participants.

The calculated CVV enabled calculating levels of perfect sync which represents the interpersonal synchrony (CVV) participants displayed when performing synchronized motion with no time difference (“lag 0”), i.e., moving in the same direction at the exact same time. Perfect sync was identified as moments of high CVV in lags of < 30 ms.

### Learning outcome measures

Two measures of learning outcome were generated based on the performance of participants at the recall session. The first involved counting the number of movements recalled in the third (recall) session. Two evaluators viewed recordings of the recall session of each learner, and counted the movements of the participants to pre-recorded, benchmark movements executed by the teachers. The judges were not aware to the learning condition (OL/IBL).

The second learning measure was based on assessing the smoothness of motion of each participant during the three sessions. This measure was shown to correspond to increased learning of motor sequences (e.g., Sosnik et al., 2004, 2014). We implemented the Gulde and Hermsdörfer (2018) Speed Metric (SM) approach that calculates a parameter that represents the ratio of average motion velocity to maximal velocity, with lower values representing smoother motion. SM values were obtained for each block in each session. First the location data-point time series provided by the Qualisys software was converted into a velocity (*v*) vector using the formula $v=\frac{\sqrt{{\left(\Delta x\right)}^{2}+{\left(\Delta y\right)}^{2}+{\left(\Delta z\right)}^{2}}}{\Delta t}$, where *Δx*, *Δy*, and *Δz* represent the difference between each two adjacent location points on the *x*, *y*, and *z* axes accordingly, and *Δt* is the inter-sample interval. Second, SM parameter was obtained as $\mathrm{SM}=\frac{\mathrm{mean}\left(v\right)}{\max\left(v\right)}$.

### Data analysis—linear mixed effects

To analyse synchrony, we used Linear Mixed Effects (LME) models, employing the LME4 package;^2^ version 1.1–27.1 for the R language (version 4.1.2). The advantage of LME models over the more common General Linear Models (GLM) approach is in that LME permit the inclusion of multiple identifiable sources of random effects, thus reducing the residual variance in the model, potentially making the model more coherent (Baayen et al., 2008). We identified two random factors in the current study: (1) the inter-personal differences between the learners, nested within their respective teacher’s identity; and (2) the otherwise unaccounted for differences between the movements comprising each block, according to the motion set to which the block belongs (i.e., block number nested within set). These were inserted into our LME models as random factors.

## Results

### Assessment of learning outcome

**Figure 3:**
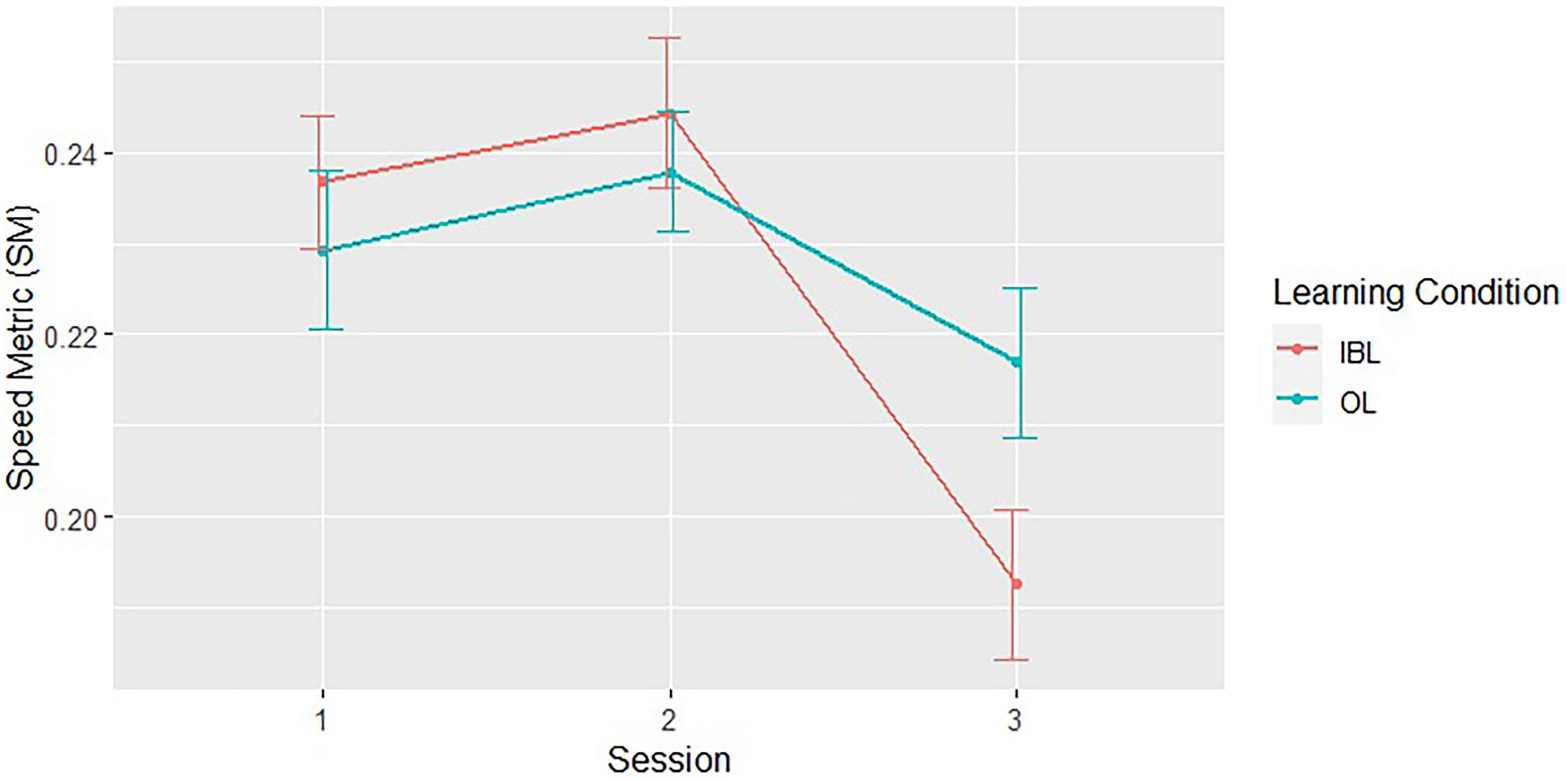
Speed Metric values at the end of the two training sessions (1–2) and the recall session (3). Error bars represent the standard error of the mean.

Motion smoothness: To compare the level of motion smoothness following each training session with the recall session, we used the SM measure from one block in each session. The 8th block was used from each of the 1st and 2nd sessions, whereas the 3rd session consisted of only one block, which was used in the analysis. Two LME models were constructed, which included SM as the dependent measure, Learning Condition and Session as fixed factors, and participant number (learner) nested within teacher’s identifier as a random factor. Here and in the following models, this nesting of the random effects was used in order to account for the possible long-term trends resulting from an increase in the teachers’ own motor skill over time, as two teachers were instructing 40 learners, with the later numbered in chronological order. The models differed in the level of interaction between these factors. Comparison of the models using a Wald type II *χ*2 test showed that the model which included the 2-way interaction between the fixed factors was most descriptive of the data [*χ*2(2) = 6.08, *p* < 0.05]. In that model, the interaction between Learning Condition and Session was significant [*F*(2, 195) = 3.06; *p* = 0.05, *η*^2^*p* = 0.03]. Analysis of this interaction showed that, in the IBL learning condition, SM was significantly lower (i.e., motion was smoother) compared to the 1st training session [*χ*^2^(1) = 17.15, *p* < 0.001], and to the second training session [*χ*^2^(1) = 25.2, *p* < 0.005], with no significant difference between the training sessions [*χ*^2^(1) = 0.63, n.s.]. In the OL learning condition, there was no significant difference in SM between recall session and the 1st training session [*χ*^2^(1) = 1.17, n.s.], or the 2nd training session [*χ*^2^(1) = 4.09, n.s.], and no significant difference between the training sessions [*χ*^2^(1) = 0.83, n.s.] (Figure 3).

**Figure 4:**
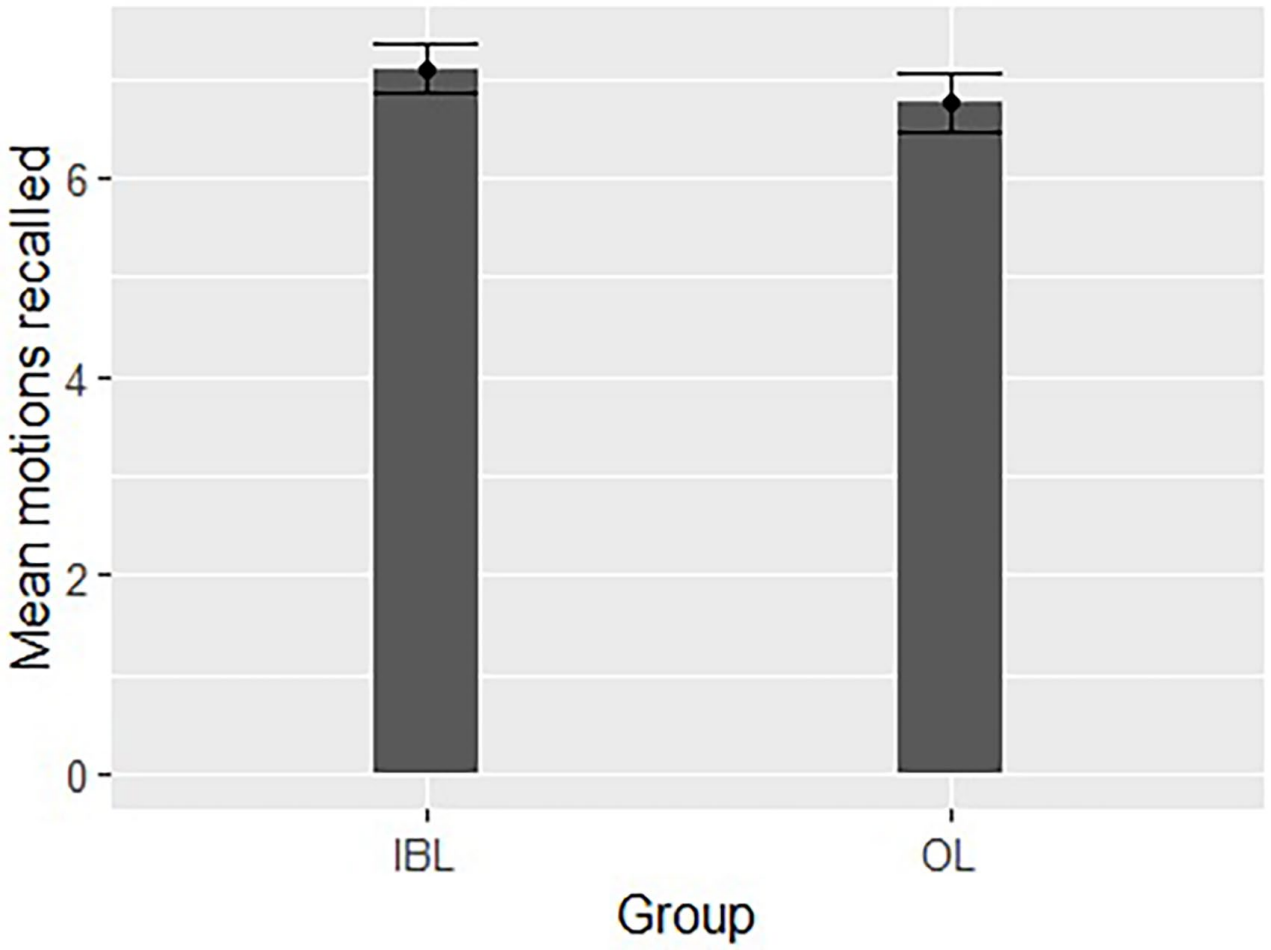
Comparison of the two learning conditions in the number of recalled movements in each learning condition. Error bars represent the standard error of the mean.

Motions recalled: We examined the overall performance following the two training sessions in the two learning conditions, using a measure reflecting the number of movements recalled. Performance scores in the recall session were compared between the OL and IBL condition groups, using an independent samples t-test of the number of correctly recalled movements for each participant in OL (*M* = 6.75, SD = 1.293) and IBL (*M* = 7.1, SD = 1.119) conditions. There was no significant effect for learning condition [*t*(38) = 0.92, *p* = 0.366] indicating that participants recalled similar number of movements in both conditions (Figure 4).

### Movement synchrony between teacher and learner

The change in motion synchrony patterns over time was assessed by comparing the first motion set of the first session to the last set of the second session. As mentioned above, each session consisted of two set of movements –A and B. In each session participants were trained in two sets, each of which consisted of movements belonging to a different set. The order of the sets was counterbalanced between participants, such that any given participant performed the sets in one of two orders: A–B → B-A, or B–A → A–B, with the first pair comprising the first session and the last two – the second session (Figure 2). We were interested in the effects of training on synchrony in sets of the same movements, and so we selected the first set of the first session and the last set of the second session for comparisons. Consequently, for each participant, the two sets that were used in the present analysis were always of the same type – either A or B.

Our model included the Perfect Sync measure as the dependent measure; learning condition (IBL/OL), set number (1, the first set; 4, the last set), and the block within each set (1 through 4 in chronological order of appearance); the random effects included group number nested within teacher’s identifier; and the motion order for each block, nested within set type (A or B). Three variations of this model were constructed: the first included only the main effects of the fixed factors; the second added all possible 2-way interactions between the fixed factors, and the third added the 3-way interaction as well. The models were compared using a Wald type II *χ*^2^ test to examine their comparative increase in predictive power vs. their respective increase in complexity. It was found that the second and third models, which included interactions, did not significantly increase in predictive power relative to the first model, which included only the main effects of the fixed factors. Therefore, the first model was used in the analyses in the following section.

**Figure 5:**
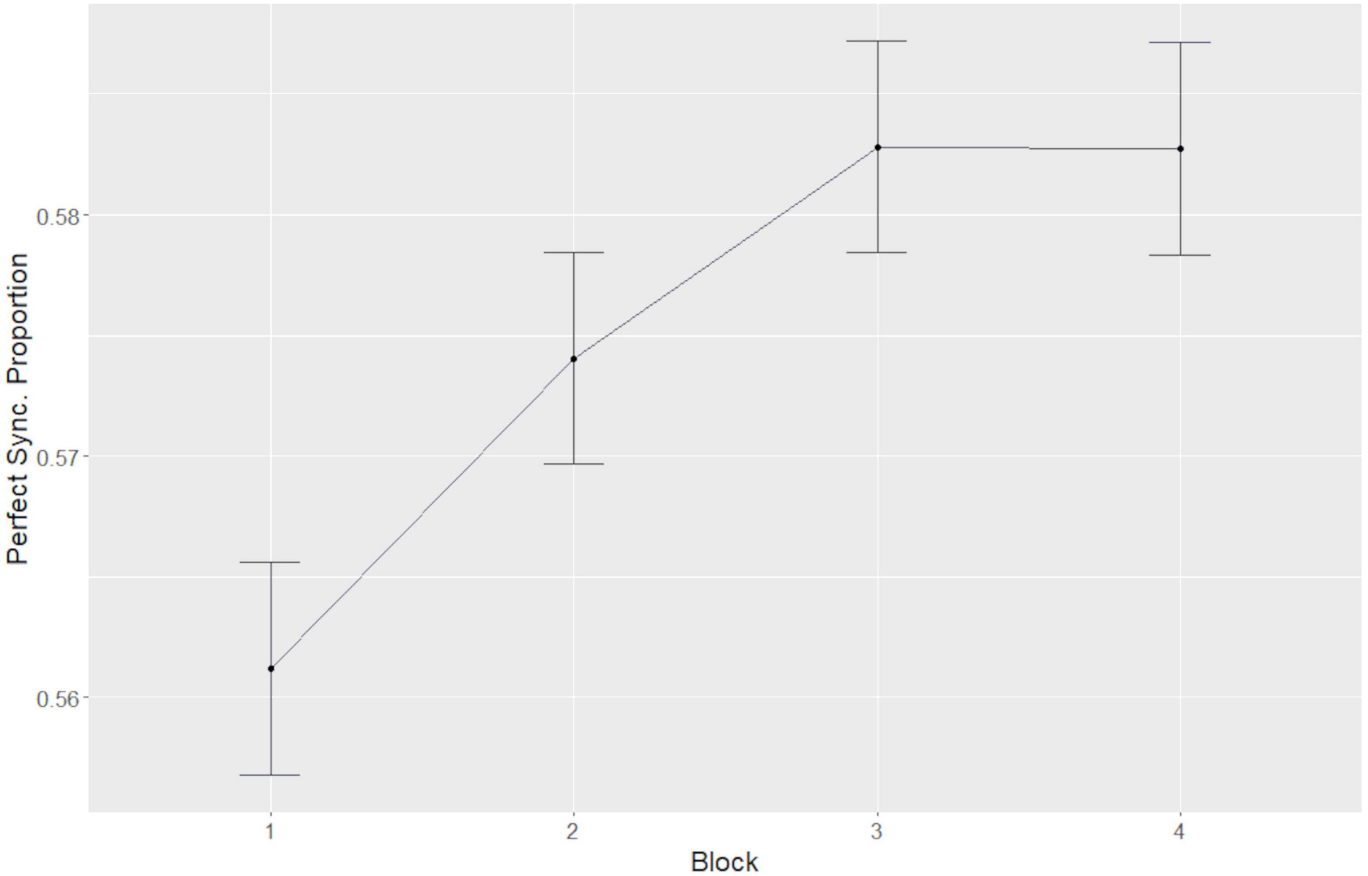
A gradual increase in synchrony throughout each motion set. Error bars represent the standard error of the mean.

Examination of the first model showed significant main effects of block [*F*(3, 570) = 10.07; *p* < 0.001, *η*^2^*p* = 0.05], such that synchrony levels increased significantly from the first block across both sets, compared to the second [*χ*^2^(1) = 14.38, *p* < 0.001], the third [*χ*^2^(1) = 23, *p* < 0.001], and the fourth [*χ*^2^(1) = 21.28, *p* < 0.001] blocks, indicating a gradual increase in synchrony throughout each motion set (Figure 5). Additionally, a significant main effect of set number [*F*(1,493) = 29.99; *p* < 0.001, *η*^2^*p* = 0.06] was found, such that mean synchrony in the first set (*M* = 0.566, SE = 0.037) was significantly lower than that in the fourth set (*M* = 0.585, SE = 0.037), indicating an overall increase in synchrony between the beginning of the first session and the end of the second session.

Despite finding no interactions of any of the fixed factors in our LME model with group type, we were interested in examining possible differences between the two groups in the simple main effects of time course within each set. Our hypothesis was that learning to synchronize should be facilitated in the IBL group, whereas no such facilitation should occur in the OL group. We have, therefore, conducted a series of planned comparisons using the third model, which included all interactions between the fixed factors.

**Figure 6:**
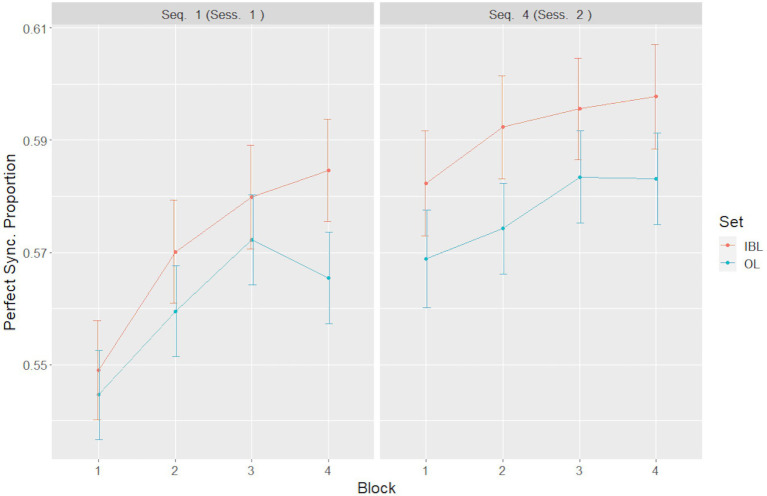
Planned comparisons of synchrony levels between blocks, sets and groups. Error bars represent the standard error of the mean.

Examination of this model, using Bonferroni correction for multiple comparisons, revealed a significant increase in synchrony [*χ*^2^(1) = 11.66, *p* < 0.05] between the first and the fourth blocks, in the first set only, for the IBL group, whereas in the OL group no significant increase in synchrony [*χ*^2^(1) = 4.66, n.s.] was detected. No significant increase was observed in either group in the fourth set, suggesting a possible saturation in synchrony training toward the end of the second session (Figure 6).

Additionally, in the IBL group, a significant increase in synchrony occurred between the first block of the first set and the first block of the second set [*χ*^2^(1) = 11.45, *p* < 0.01], whereas the increase in the OL group was not significant [*χ*^2^(1) = 5.82, n.s.], indicating that while the IBL group started the second session with a synchrony level that was significantly higher than the initial level at the onset of the first session, the same did not reach significance in the OL group. When we analysed the difference between the 4th block of the first session and the 1st block of the second session, we found a significant main effect of learning condition [*F*(1, 29) = 5,47; *p* < 0.05, *η*^2^*p* = 0.16], such that the synchrony level in the IBL condition was higher than that in the OL condition. No other main effects or interactions were found.

In order to provide a complete picture of the results, we have conducted a similar analysis, but included all four sets in our model. Similarly to the previously reported results, the one-way model was found to provide the best fit for the data. Again, the main effect of of block was significant [*F*(3, 1,184) = 7.4; *p* < 0.001, *η*^2^p = 0.02], such that synchrony levels increased significantly from the first block across both sets, compared to the second [*χ*^2^(1) = 9.76, p < 0.01], the third [*χ*^2^(1) = 17.84, *p* < 0.001], and the fourth [*χ*^2^(1) = 14.88, *p* < 0.001] blocks. The main effect of set number was, likewise, significant [*F*(3, 1,184) = 10.43; *p* < 0.001, *η*^2^*p* = 0.03], indicating an increase in synchrony over blocks. No significant main effect of learning condition was found [*F*(1, 36) = 2.22; n.s.]

### Movement synchrony as a predictor of learning

We explored the possibility that the levels of synchrony observed in the 1st and the 2nd sessions was a direct predictor of motion smoothness in the 3rd session. To examine this, an LME model was constructed with SM (in the recall session) as the predicted measure, Learning Condition and Perfect Sync. (in the learning sessions) as fixed factors, and Participant as a random factor. However, none of the main effects or interactions of the fixed factors in this model reached statistical significance.

## Discussion

The aim of the study was to examine the advantage of interaction-based learning compared to observational learning in the context of movement synchronization. In contrast to previous studies that examined individual learning, we focused on learning in interactions that require mutual predictions and bidirectional coordination of movements. Furthermore, while previous studies have used motor paradigms that are kinematically simple, here we tested 3D motion, which is closer to complex real-life movements and involve coordination of multiple joints and body segments.

We examined the difference in learning outcomes between two learning groups: OL, in which the teacher could not see the learner, and IBL in which the teacher and learner could see each other. The results show that although the number of movements recalled was similar in the recall session in both groups, learners in the IBL group showed increased movement smoothness as compared to learners participating in the OL group. Movement smoothness characterizes movements that are performed in a continual fashion without any interruptions or jitters. Smooth movements are a characteristic feature of healthy and well-trained motor behavior (Sejnowski, 1998). Non-smooth action is characterized by jittery movement and is thought to originate from a reactive learner that adjusts the movement based on the perceived tracking error (Noy et al., 2011). On the other hand, movement smoothness increases with motor learning (Sosnik et al., 2004, 2014), and is considered to be the result of effort minimization (Burdet et al., 2013)—a central representation of motor learning (Franklin et al., 2008). Thus, increased smoothness in the IBL group may indicate that the mutual feedback between the teacher and learning in this group allowed the learner to generate a more accurate internal models of the sequences during the acquisition stage. The consolidation of these models then facilitates performance at subsequent stages.

As predicted, a significant improvement was found in the amount of time the teacher and learner moved in perfect synchrony between the two sessions in both learning groups. Notably, a significant, gradual improvement in perfect synchrony during the first session was evident only in the IBL group, indicating that bidirectional feedback allows participants to increasingly improve their ability to coordinate their moments with the teacher. It is important to note that although there were no group differences in synchrony, a significant improvement was found in the amount of time the teacher and learner moved in perfect synchrony in the first session, in the IBL group but not in the OL group. Similar changes were not present in the second session of the IBL group. In comparison, the OL group did not show any significant improvement. While overall perfect synchrony levels were observed within a motion set, as well as between sessions, a closer examination of this phenomenon revealed that both effects were present in the IBL, but not in the OL learning condition. Specifically, the IBL group exhibited a significant gradual increase in synchrony during the first set, which resembles a learning curve. Furthermore, the IBL group started the last set at a synchrony level that was significantly higher than the initial level at the start of the first set. Conversely, neither effect reached significant levels in the OL group: There was no significant increase in synchrony in the first set, and the last set did not start at significantly higher synchrony level as compared to the starting point. This confirms our hypothesis that IBL, rather than OL, permits motion synchronization to be learned and retained over time. These findings are in line with Okazaki et al. (2015) who found that it is essential to have mutual visual feedback for synchronization to emerge. Fox et al. (2017) have demonstrated that motor learning requires consolidation and repetition. The term ‘savings’ represents a condition where relearning of the sequence is more rapid than the original learning, pointing to the consolidation of motor learning (Krakauer and Shadmehr, 2006). In our experiment, the retention of synchronization in the second session in the IBL group may point to savings of the movement synchronization learning in the second session. A possible explanation for the advantage of IBL is that the mutual feedback allows the teacher to perform the movement sets in a more attuned and ‘personalized’ manner allowing the learner better adjustment to the teacher.

Although we found that synchrony increased during the training sessions, we did not find overall group differences in synchrony, indicating similar levels of movement synchrony between the IBL and OL groups. It is possible that the learned movements were not complex enough and reached a ceiling effect. The lack in complexity can arise from either the duration of each movement, or each movement’s motor components. It was previously suggested that movement duration of about 10–20 s during a continuous learning session of Tai-Chi (a similar duration of the movements in our study) is recommended for memory gains and motor learning (Tao et al., 2016; Tubytė et al., 2018). Therefore, it may be assumed that the movements’ level of difficulty was not due to their duration, but to the simplicity of the movements’ motor components. Furthermore, there were no significant differences in the spontaneous recall of movements between OL and IBL. It is possible that more difficult movements, which would demand higher levels of concentration, practice, and engagement, might further emphasize the difference in performance between the two groups. Therefore, future research should take the contribution of movement difficulty into consideration.

Previous models of motor learning view motor skill learning as a three-phase process (Fitts and Posner, 1967), consisting of an initial cognitive phase involving explicit attempts to learn the skill, followed by an associative phase in which the chosen strategy for the motor skill is refined. In the final phase, as an internal model of the skill is acquired, the cognitive explicit involvement diminishes while the skill becomes automated. The cognitive phase requires a high level of cognitive activity to process the incoming sensory information, and to produce the movement sequences. As social interactions involving support were previously found increase activity in regions related to cognitive regulation (Korisky et al., 2020), it is possible that during learning movement sequences in interactions, the cognitive phase may be enhanced. With practice (from the associative phase onwards), the dependence on the teacher diminishes and accuracy may increase.

In terms of the brain networks that are involved, the observation-execution (mirror neurons) system likely plays a critical role in IBL. The observation-execution system is typically active during both the execution of an action, and during the observation of action (Rizzolatti and Sinigaglia, 2016). Therefore, during synchronization this system may be active in both partners when trying to coordinate and imitate each other’s action. During early learning, the learner copies and executes the movement sequences produced by the teacher. The teacher, on the other hand, adapts her movements to the learner (e.g., varying the velocity of her movement). Gradually, the teacher and learner develop internal representations of each other. The teacher learns the characteristics of the learner’s movement and the learner learns the characteristics of the teacher’s movements. At this point, the learner may execute an entire sequence in a predictive manner, without reliance on continuous sensory input.

A limitation of the current study is the relatively small number of participants. Although 20 participants per condition is the minimal number of participants for a behavioral study in humans, a higher number of participants per group is recommended for greater statistical power. Another important limitation is the number of sessions. A higher number of sessions may be beneficial for learning, and will allow testing more complex tasks, such as recalling the order in which the movements were presented in the task. In addition, we did not debrief the learners in the OL condition as to whether they were aware of the unidirectional function of the mirror, and that the teachers were unable to see them. This may have affected they perception of the learning process differently from the IBL group. We suggest that future studies explicitly address this issue, for example, by having separate informed and uniformed groups in the OL condition. Finally, the inconsistency of the time intervals between sessions can cause a difference in learning. Keeping a fixed time interval throughout the experiment may be crucial to the establishment of retention and consolidation of learning.

To the best of our knowledge, our study is the first to test how social interaction affects learning of movement sequences. This aspect of learning has become especially relevant in recent years, since remote online learning in general, and online motor learning specifically, became prevalent due to the outbreak of the COVID-19 pandemic. The paradigm proposed in this study offers a new approach for understanding the effects of remote learning under controlled settings. Our study also presented a novel physical setup – window vs. one sided mirror—that allows comparing IBL and OL with a high level of control over intervening environmental variables. Furthermore, our study is the first to examine synchrony retention using classical learning curves, and examining their properties for different learning conditions. This method of analysis could be applied in future research of brain-to-brain coupling, and its relationship with motor synchrony and learning.

## Data availability statement

The raw data supporting the conclusions of this article will be made available by the authors, without undue reservation.

## Ethics statement

The studies involving human participants were reviewed and approved by Psychology Department, University of Haifa. The patients/participants provided their written informed consent to participate in this study. Written informed consent was obtained from the individual(s) for the publication of any potentially identifiable images or data included in this article.

## Author contributions

AM contributed to the data interpretation, data analysis, and wrote the manuscript. GN led the overall study, contributed to the data collection and interpretation, and wrote the manuscript. RB conceived and designed the experiment setup and contributed to the data analysis. SS-T supervised the study, reviewed and edited the manuscript. All authors contributed to the article and approved the submitted version.

## Funding

The publication of this paper was supported by European Research Council (ERC) under the European Union’s Horizon 2020 Research and Innovation Programme (grant agreement no. INTERPLASTIC: 101020091; DLV-101020091) and a grant from the Israel Science Foundation (ISF) grant 959/18.

## Conflict of interest

The authors declare that the research was conducted in the absence of any commercial or financial relationships that could be construed as a potential conflict of interest.

## Publisher’s note

All claims expressed in this article are solely those of the authors and do not necessarily represent those of their affiliated organizations, or those of the publisher, the editors and the reviewers. Any product that may be evaluated in this article, or claim that may be made by its manufacturer, is not guaranteed or endorsed by the publisher.

## Footnotes

^1^Permit#201/20

^2^ https://cran.r-project.org/web/packages/lme4/lme4.pdf
